# Malaria

**Published:** 1901-03-16

**Authors:** 


### Hospital Clinics and Medical Progress.

### MALARIA.

The March number of the *Practitioner* is devoted almost entirely to the subject of malaria, and although the story of the parasite has been so frequently retold of late as to have become a little trite it can never be without interest. Dr. Sambon gives a very good account of the paroxysms of pyrexia, with their cold, and hot, and sweating stages, which characterise the intermittent fevers. These paroxysms are due to the presence of certain hæmatozoa in the blood, and they indicate a certain stage in the development of these organisms. At the present day we know of four different types of fever corresponding to four different species of parasites, these four types being known as tertian, quartan, semi-tertian or "æstivo-autumnal fever," and quotidian. We need not here enter into the difference between these fevers, but we may mention that while each of the several types mentioned evidently depends upon a different parasite a considerable number of abnormal cases occur, some of which seem to be due to double or multiple infection, either by different groups of parasites of the same species, overlapping in point of time, or by entirely different species. Let us now turn back to the life history of the parasite as described by Dr. Rees, premising that although the form taken by the parasite in certain stages of its growth differs in the several species, the various metamorphoses it undergoes are practically the same in all. The malaria organism is now universally regarded as belonging to the class "Sporozoa." It is parasitic in man and in a certain genus of mosquito (Anopheles): the former is its intermediate host, and the latter its definitive host. It has two modes of reproduction: one, which takes place within the human red blood corpuscle, is by spore formation and is an asexual process. This can be carried on and attain maturity, and be repeated again and again, without the intervention of the mosquito. This asexual process was first carefully described by Golgi, and may be spoken of as the cycle of Golgi; and it is this cycle that has to do with the production of the symptoms of the disease. The other cycle is a sexual process, which can only be completed by passage through a certain genus of mosquito, and our knowledge of this sexual cycle being principally due to the researches of Ross, it may be called the cycle of Ross. While then by aid of the cycle of Golgi the parasite can continue its life for considerable periods within man's blood, by aid of the cycle of Ross it can be carried from man to man and thus perpetuated. In the early stages of the development of the parasite in the red blood cell there seems nothing to show whether it will undergo the prolonged and roundabout sexual form of reproduction *via* the mosquito, or whether it will take the short circuit within the blood by asexual spore formation. But at a certain point a difference is to be noted in certain of the parasites. Instead of continuing to fill up the blood corpuscle, becoming sporocytes, and ultimately breaking up into a host of spores, which again in turn enter fresh blood corpuscles—an asexual cycle which may be completed in from one to three days—certain parasites undergo further development, forming in the benign fevers large pigmented spheres, and in the case of the malignant fevers the well-known crescent bodies, which when swallowed by the mosquito develop into male and female forms (gametes). The fertilised females undergo several changes, ultimately rupturing and throwing into the body fluid of the mosquito a large number of sickle-shaped bodies (sporozoites) which are in the end carried to the salivary glands. It is these bodies which are the actual source of infection to man: they have been traced as far as the proboscis of the mosquito, and when the mosquito "bites," these sporozoites enter man's blood and attack the red blood corpuscles just as the spores formed in the Golgi cycle do, and thus the process commences over again.

What then is the relation between the several phases in the development of the parasite and the different stages of the disease? Dr. Sambon says that by examining at short intervals the blood of patients suffering from simple tertian or simple quartan fever we soon learn that the respective parasites of these fevers infect the blood in great groups made up of thousands of organisms, all at approximately the same stage of development, and that there is a constant definite correlation between the different manifestations of each fever and the various developmental stages of its specific parasites. During the cold stage, and immediately before its onset, we find the parasites fully developed and in process of segmentation; we see the segments or young parasites emerging from the disintegrating corpuscles, and we see phagocytes carrying off to the internal eliminative organs pigment granules, segmentation residua, and parasites. Throughout the hot stage we find the young organisms attacking the erythrocytes, and we can see them still adhering to the corpuscle or just safely inside; while at the end of the paroxysm all the young protozoa that have not been able to take immediate shelter inside normal erythrocytes have been engulfed and carried off by the phagocytes. During the intermission we can watch the progressive development of the endo-globular parasites, and we can see them actively absorbing the cell contents. The excrementitious part of the ingested hæmoglobin forms the characteristic granules of black pigment which are usually distributed about the periphery of the hæmatocytozoa. Finally, a few hours before the onset of the next paroxysm, we find all the parasites preparing to renovate their youth by breaking up into a number of segments. Thus we gather that this segmentation occurs almost simultaneously in all the members of a group of parasites just as plants flower at the same time, and that the fever paroxysm begins just at the time of complete segmentation, when the young parasites are swarming in the *liquor sanguinis*, and terminates when all the organisms of the new brood have either penetrated into the red corpuscles or have been destroyed by the phagocytes.

Dr. Sambon believes that the pyrexia has to do with the phagocytosis rather than with the formation of any toxic substance at the moment of segmentation, and he says that a high pyrexia is usually followed by marked improvement, while on the other hand the most severe cases are those characterised by an enormous number of parasites and hardly any reaction. In semi-tertian and quotidian fevers the same sequence takes place, but we cannot demonstrate it so satisfactorily because the segmentation of these parasites takes place within the internal organs, and only a few in the early endoglobular stages are usually to be found in the peripheral circulation. Moreover the segmentation is spread over a longer period, which perhaps has to do with the longer duration of the paroxysms of the semi-tertian and quotidian fevers.

Much discussion has taken place as to whether man is the only alternate host of the malaria parasite. It is admitted that it is only in the body of the mosquito that the parasite can pass through the sexual form of reproduction which is essential to the perpetuation of the species, but it is not quite so clear that human blood is the only blood which will serve as its intermediate host. Lately it has been the fashion to speak very positively on this subject, and several authors have laid it down as a thing no longer to be questioned that the malaria organisms are peculiar to human blood, and that their alternate phases of existence oscillate exclusively between man and a certain genus of mosquito. On this theory has been founded the suggestion that failing to kill all the mosquitoes—a hopeless task—it might be possible to put a stop to malaria by systematically attacking the parasite in man by means of quinine, and thus preventing the possibility of the mosquitoes becoming infected. Well, it may be so; but to state that it is so involves accepting a negative as proven, and we are glad to find Dr. Patrick Manson speaking somewhat cautiously on the subject. He says: "There can be little doubt that the principal and usual source from which the mosquito derives the malaria parasite is man; but to assert, as some have done, that man is the only source is, I consider, going beyond the evidence. If in such a matter analogy be accorded weight, the contrary might very well be the case. We know that the corresponding avian hæmamœbidæ, proteosoma and halteridium, each of them affects several bird-hosts. This fact might well be held to indicate that the hæmamœbidæ of man also have a plurality of vertebrate hosts. . . . We know that similar parasites have been found in monkeys, dogs, sheep, horses, and oxen, so that man is not the only mammal capable of harbouring the genus. Dionisi . . . has shown that of three species of bat each harbours a special species of parasite resembling one or other of the human hæmamœbidæ. . . . On the assumption that man is the only intermediary of the parasites it is difficult to understand the insalubrity of many very sparsely populated districts, and also the sudden explosion and rapid spread over large areas of malarial epidemics. These things are more comprehensible on the assumption that some other animal may share with man the privilege of being the intermediary of the malaria parasite."

